# Association between social relationship of mentors and depressive symptoms in first-time mothers during the transition from pregnancy to 6-months postpartum

**DOI:** 10.1186/s40748-024-00175-7

**Published:** 2024-02-02

**Authors:** Malgorzata Gasperowicz, Karen M. Benzies

**Affiliations:** 1 Faculty of Nursing, University of Calgary, PF3280C - 2500 University Drive NW, Calgary, AB T2N 1N4 Canada; 2 Departments of Pediatrics and Community Health Sciences, Cumming School of Medicine, University of Calgary, Calgary, AB Canada; 3 Alberta Children’s Hospital Research Institute, Calgary, AB Canada

**Keywords:** First-time mothers, Postpartum, Depressive symptoms, Mentorship, Social support

## Abstract

**Background:**

First-time motherhood is characterized by high psychosocial distress, which untreated, has serious consequences. Informal social support provided by specially trained mentors may be protective against postpartum depressive symptoms but may vary by women’s social relationship with the mentor. The objective of this study was to evaluate the association of types of mentors on women’s depressive symptoms between late pregnancy to 6-months postpartum and the characteristics of women associated with mentor type.

**Methods:**

This study was a secondary analysis of data from a community sample of 312 primiparous women from a single-group, longitudinal intervention study of Welcome to Parenthood. Welcome to Parenthood provided education and mentorship for women during the transition from pregnancy to postpartum. Women completed the Edinburgh Postnatal Depression Scale (EPDS) in late pregnancy, and 2- and 6-months postpartum.

**Results:**

Women who recently relocated were less likely to be mentored by their mothers and more likely to be mentored by friends or volunteers. Women who were mentored by their mothers or sisters scored the lowest on the EPDS; those mentored by their mothers-in-law scored the highest. Women who were mentored by other family, friends, or volunteers scored between the two extremes. EPDS scores of women mentored by each type of mentor decreased from pregnancy to 6-months postpartum; only for mother-, sister-, and volunteer-mentored groups was this decrease significant.

**Conclusions:**

During transition to parenthood, support provided by mothers or sisters is best for women’s mental health but may not always be available to women who have recently relocated. In such situations, specially trained community volunteers may be the second-best option.

## Background

For women, the transition from pregnancy to early parenthood involves a shift in family dynamics, sleep disruption, fatigue, social isolation, new role identity, and heightened psychosocial stress [1–3]. Maternal mental health is crucial during early parenthood. Globally, approximately 17% of mothers suffer from minor or major postpartum depression (PPD; 4). PPD symptoms [5], including persistent subclinical ones [6], are associated with behavior problems, insecure attachment, lower cognitive development, and poor infant growth [6–8]. Maternal low socio-economic status, lifestyle (e.g., smoking, alcohol use), and infant characteristics (e.g., preterm birth) were associated with higher rates of PPD [4].

Suboptimal social support is a predictor of antenatal [9] and postpartum depression [4]. We define social support as, “social transactions that are ‘perceived by or intended by the provider to facilitate coping in everyday life, and especially in response to stressful situations’” (p. 6) [10]. Social support is positively associated with parenting outcomes, including infant care, adjustment to the maternal role, self-esteem, and lower stress [11]. In a systematic review and meta-analysis, peer support from another mother who had previous experience with PPD reduced maternal antenatal and postpartum depressive symptoms and risk of depression [12].

Mentorship is a form of social support where the mentor is an experienced, trusted individual who provides guidance, motivation, emotional support, and role modeling for the mentee [13]. When mentorship is formalized, it includes clear expectations for frequency of encounters, length of commitment, and strategies to enhance social support. An integrative literature review reported that volunteer social support (or mentorship) programs can improve maternal mental health, parenting skills, parent-child relationships, and social capital [14]. There are few evidence-based mentorship programs to support mothers between pregnancy and early parenthood, and most focus on vulnerable populations (e.g., 15, 16). Mitchell et al. [16], in a qualitative study of Mentoring Mums, a program that bridged the gap between mothers and professional support, conceptualized the role of volunteer mentors as “befriending” (p. 40) and building a network of connections for young mothers at risk for poor parenting. Grace et al. [15], in their seven-site randomized controlled trial of the Volunteer Family Mentor program for vulnerable and isolated families, reported a group difference in parenting sense of competence favoring the intervention group, but no group differences on maternal mental health. In a systematic review of the effects of support from the infant’s grandparents, Riem et al. [17] found that support from maternal grandmothers constituted a protective factor against the development of postnatal mental health problems in the mother.

Welcome to Parenthood™ (W2P), an intervention for women in the transition from pregnancy to parenthood, reduced depressive symptoms and improved infant development [18]. W2P consists of (a) parenting education, (b) mentorship, and (c) an engagement tool [18]. Explained briefly, W2P facilitators from participating agencies schedule a 2-hour group parenting education session. To ensure the mother, father, and mentor receive similar information, they attend the same session. Mentors learn how to provide informal social support to the mother during a minimum of 20 contacts and when to reach out for help from a professional when they recognize depressive symptoms. Given expectant parents want or need essential items for a newborn, a Baby Kit containing some essentials is provided to each family. While women reported fewer depressive symptoms, it was unclear whether the social relationship between woman and mentor influenced these symptoms.

The objective of the current study was to explore whether the type of social relationship (e.g., family, friends, or volunteers) between first-time mothers and mentors trained in W2P influenced depressive symptoms. The research questions were: (a) What is the association between the type of social relationship (woman and mentor) and the trajectory of depressive symptoms from pregnancy to 6-months postpartum? and (b) What socio-demographic factors in the woman are associated with mentor type?

We used Stewart’s [10] social support theory to this study. Accordingly, social support should be considered by (a) disposition—the recipient’s appraisal of pre-existing social embeddedness of connections; (b) duration—durability or stability of social support; and (c) direction—unidirectional support without expectation of reciprocity (typical of professional support), or bi-directional with implied reciprocity (typical for informal support).

## Methods

### Design and setting

This study is a secondary analysis of data from the W2P study [18]. We used the Transparent Reporting of Evaluations with Nonrandomized Designs (TREND) checklist [19]. Briefly, we used a single-group, longitudinal design. Between October 2015 and August 2017, we collected data in Alberta, Canada, which has a demographically diverse population of 4.23 million and a publicly funded health care system [20]. We implemented W2P through 11 family resource centers that offered parenting programs and supports for healthy child development. The study was approved by The University of Calgary, Conjoint Health Research Ethics Board (REB 14–0557).

### Participants and procedures

We recruited women through prenatal classes, community health and medical clinics, and Facebook [18]. Women and their mentors provided informed, written consent to participate in this study. Women were eligible if they were: (a) 18 years or older, (b) able to communicate in English, (c) willing to attend a 2-hour parenting education session, (d) between 30 and 34 weeks gestation with their first child, and (e) willing to be mentored until their infant was 6 months old. Women identified a mentor from their own family or social network. If unable to identify a mentor, the family resource center identified a mentor for her. Mentors were eligible if they satisfied criteria (a), (b), and (c) listed above and were: (d) willing to mentor a first-time mother from the third trimester of pregnancy until the infant was 6 months old, and (e) willing to write responses to standardized questions in a specially designed journal after each contact with their mentee and complete a questionnaire when the infant was 6 months old. Participants completed paper surveys in-person or via telephone. W2P facilitators entered data into a web-based platform. We collected data during the last trimester of pregnancy, and at 2-and 6-months postpartum.

### Measurement

The Edinburgh Postnatal Depression Scale (EPDS; [21]) is the most used pre- and postnatal depression screening tool, with 10 self-report items. The theoretical range of scores is 0 to 30; higher scores indicate more depressive symptoms. Cut-offs that indicate likely major depressive disorder vary between 10 and 13. In our study, a score ≥ 10 or a positive response to the suicidal ideation item triggered a follow-up that included providing the first-time mother with a referral letter for her physician or public health nurse. An investigator-designed questionnaire was used to collect demographic and health information and type of social relationship between the mother and her mentor.

### Data analyses

We analyzed data with SPSS 27.0 (IBM Corp. Released 2020. IMB SPSS Statistics for Windows, Version 27.0. Armonk, NY: IBM Corp) and GraphPad Prism 6.0 (GraphPad Prism version 8.0.0 for Windows, GraphPad Software, San Diego, CA: GraphPad). Given multiple comparisons in this study, we applied a Bonferroni correction (0.05/9) with the *p* value set at 0.006 to indicate statistical significance. We described participant characteristics using means/standard deviations and frequencies/percentages. To compare women’s characteristics by mentor type, we used χ^2^ tests and Adjusted Residuals (AR) as post hoc comparisons [22]. Within most mentor types, EPDS scores were not normally distributed; therefore, we used a Kruskall-Wallis test to compare EPDS mean scores between mentor type at the same time point. We compared EPDS mean scores between enrollment and 6-months postpartum for each mentor type using a Wilcoxon two-tailed matched pairs signed ranked *t*-test.

To evaluate the time trajectories of maternal depressive symptoms for each mentor type, we fit EPDS mean scores to straight lines using linear regression. To test whether the EPDS mean score trajectories were different between the six mentor types, we used a built-in GraphPad Prism method (https://www.graphpad.com/guides/prism/latest/curve-fitting/reg_comparingslopesandintercepts.htm). Briefly, Prism calculates a *p*-value (two-tailed) testing the null hypothesis that the slopes are identical. If slopes are not significantly different, Prism calculates a single slope for all the lines and calculates a second *p*-value, testing the null hypothesis that the lines are identical using the *F*-test.

## Results

### Characteristics of women and mentors

In the original W2P study [18], of the 554 women who met eligibility criteria and completed an enrollment questionnaire, 454 completed a 2- and/or 6-month follow-up questionnaire. In this current study, we included 312 women who identified the type of social relationship to the mentor. See Table 1 for participant characteristics.

**Table 1:** Characteristics of Mothers and Mentors (*N* = 312)

	Mothers	Mentors
Characteristic	*n* (%)	*n* (%)
**Age**		
< 25	45 (14.5)	7 (2.2)
25–34 years	225 (72.6)	105 (33.7)
35–44 years	39 (12.5)	67 (21.5)
45–54 years	1 (0.3)	51 (16.3)
55–64 years	0 (0.0)	59 (18.9)
≥ 65	0 (0.0)	15 (4.8)
Not reported	2 (0.6)	8 (2.6)
**Highest Level of Education**		
High school diploma or less	40 (12.8)	51 (16.3)
Certificate/diploma after high school	74 (23.7)	93 (29.8)
College/university degree	198 (63.5)	167 (53.5)
Not reported	0 (0.0)	1 (0.3)
**Employment Status**		
In paid employment	216 (69.2)	201 (64.4)
Not in paid employment	39 (12.5)	51 (16.3)
Maternity/sick leave/leave of absence	56 (17.9)	24 (7.7)
Retired or on disability	0 (0.0)	35 (11.2)
Not Reported	1 (0.3)	1 (0.3)
**Household Annual Income**		
< $40,000	30 (9.6)	34 (10.9)
$40,000 - $79,999	46 (14.7)	55 (17.6)
≥ $80,000	198 (63.5)	172 (55.1)
Not reported	38 (12.2)	51 (16.3)
**Marital Status**		
Partnered	292 (93.6)	259 (83.0)
Not partnered	18 (5.8)	50 (16.0)
Not Reported	2 (0.6)	3 (1.0)
**Born in Canada**		
Yes	252 (80.8)	262 (84.0)
No	60 (19.2)	48 (15.4)
Not Reported	0 (0.0)	2 (0.6)
**Time Lived in Alberta**		
< 5 years	74 (23.7)	32 (10.3)
≥ 5 years	233 (74.7)	270 (86.5)
Not Reported	5 (1.6)	10 (3.2)
**Ethnicity**		
White	240 (76.9)	260 (83.3)
Indigenous	13 (4.2)	13 (4.2)
Visible minorities	59 (18.9)	36 (11.5)
Not Reported	0 (0.0)	3 (1.0)
**Language Spoken at Home**		
English	285 (91.3)	287 (92.0)
Other	27 (8.7)	24 (7.7)
Not Reported	0 (0.0)	1 (0.3)

On average, women were 29.5 ± 4.6 (range 18–47) years old and mentors were 43.1 ± 12.9 (range 19–77) years old. There were six types of mentor relationships to the woman: own mother (*n* = 93), mother-in-law (*n* = 16), sister (*n* = 23), other family member (*n* = 32), friend (*n* = 102), or community volunteer (*n* = 48). All mentors were female. See Table 2 for characteristics of women by mentor type.

**Table 2:** Characteristics of Mothers for Full Sample and by Type of Mentor (*N* = 312)

Characteristics	Mother *n* = 93	Mother-in-Law *n* = 16	Sister *n* = 23	Other Family Member *n* = 30	Friend, Roommate, Coworker *n* = 102	Volunteer *n* = 48		Full Sample *N* = 312
	***n*** **(%)**	***n*** **(%)**	***n*** **(%)**	***n*** **(%)**	***n*** **(%)**	***n*** **(%)**	***p*** ^e^	***n*** **(%)**
**Age** ^a^							0.040	
< 25	19 (20.7)	4 (25.0)	0 (0.0)	6 (20.0)	6 (5.9)	10 (20.8)		45 (14.5)
25–34 years	64 (69.6)	11 (68.8)	21 (91.3)	20 (66.7)	78 (77.2)	31 (64.6)		225 (72.6)
≥ 35 years	9 (9.8)	1 (6.3)	2 (8.7)	4 (13.3)	17 (16.8)	7 (14.6)		40 (12.9)
**Education**							0.080	
High school diploma or less	11 (11.8)	3 (18.8)	3 (13.0)	10 (33.3)	7 (6.9)	6 (12.5)		40 (12.8)
Certificate/diploma after high school	24 (25.8)	3 (18.8)	5 (21.7)	5 (16.7)	23 (22.5)	14 (29.2)		74 (23.7)
College/university degree	58 (62.4)	10 (62.5)	15 (65.2)	15 (50.0)	72 (70.6)	28 (58.3)		198 (63.5)
**Employment** ^b^							0.049	
In paid employment	72 (77.4)	12 (75.0)	19 (82.6)	16 (53.3)	72 (70.6)	25 (53.2)		216 (69.5)
Not in paid employment	9 (9.7)	2 (12.5)	1 (4.3)	8 (26.7)	12 (11.8)	7 (14.9)		39 (12.5)
Maternity/sick leave/leave of absence	12 (12.9)	2 (12.5)	3 (13.0)	6 (20.0)	18 (17.6)	15 (31.9)		56 (18.0)
**Household Income**							0.180	
< $40,000	8 (8.6)	3 (18.8)	0 (0.0)	2 (6.7)	9 (8.8)	8 (16.7)		30 (9.6)
$40,000 - $79,999	14 (15.1)	3 (18.8)	2 (8.7)	8 (26.7)	14 (13.7)	5 (10.4)		46 (14.7)
≥ $80,000	59 (63.4)	8 (50.0)	18 (78.3)	16 (53.3)	72 (70.6)	25 (52.1)		198 (63.5)
Not reported	12 (12.9)	2 (12.5)	3 (13.0)	4 (13.3)	7 (6.9)	10 (20.8)		38 (12.2)
**Marital Status** ^c^							0.058	
Partnered	87 (93.5%)	15 (93.8%)	22 (100.0%)	30 (100.0%)	97 (96.0%)	41 (85.4%)		292 (94.2%)
Not partnered	6 (6.5%)	1 (6.3%)	0 (0.0%)	0 (0.0%)	4 (4.0%)	7 (14.6%)		18 (5.8%)
**Born in Canada**							**< 0.001**	
Yes	87 (93.5%)	13 (81.3%)	22 (95.7%)	25 (83.3%)	73 (71.6%)	32 (66.7%)		252 (80.8%)
No	6 (6.5%)	3 (18.8%)	1 (4.3%)	5 (16.7%)	29 (28.4%)	16 (33.3%)		60 (19.2%)
**Time in Alberta** ^d^							**< 0.001**	
< 5 years	6 (6.5)	3 (18.8)	2 (9.1)	8 (26.7)	32 (32.0)	23 (48.9)		74 (24.1)
≥ 5 years	86 (93.5)	13 (81.3)	20 (90.9)	22 (73.3)	68 (68.0)	24 (51.1)		233 (75.9)
**Ethnic Background**							0.066	
White	81 (87.1)	12 (75.0)	19 (82.6)	21 (70.0)	73 (71.6)	34 (70.8)		240 (76.9)
Indigenous	5 (5.4)	1 (6.3)	0 (0.0)	3 (10.0)	3 (2.9)	1 (2.1)		13 (4.2)
Visible minorities	7 (7.5)	3 (18.8)	4 (17.4)	6 (20.0)	26 (25.5)	13 (27.1)		59 (18.9)
**Language at Home**							**< 0.001**	
English	92 (98.9%)	16 (100.0%)	23 (100.0%)	28 (93.3%)	86 (84.3%)	40 (83.3%)		285 (91.3%)
Other	1 (1.1%)	0 (0.0%)	0 (0.0%)	2 (6.7%)	16 (15.7%)	8 (16.7%)		27 (8.7%)

*Note*. Due to missing values, sample size varies: ^a^ age = 310; ^b^ employment = 311; ^c^ marital status = 310; ^d^ time lived in Alberta = 307. ^e^ Pearson χ^2^ with Bonferroni correction significant *p*-values < 0.006 in bold

There were no significant differences in women’s age, employment, education, household income, or marital status by mentor type. There were significant differences in women’s migratory status and language spoken at home by mentor type. Using ARs for χ^2^ as post hoc tests, women born in Canada (AR = 3.7), lived in Alberta more than 5 years (AR = 4.7), and spoke English at home (AR = 3.1) were more likely to be mentored by their own mother (*p* <.001). Women not born in Canada (ARs = 2.9 and 2.7), lived in Alberta fewer than 5 years (ARs = 2.2 and 4.3), and spoke a language other than English (ARs = 3.1 and 2.1) were more likely to be mentored by a friend or volunteer, respectively (*p* <.001).

### Decrease in depressive symptoms over the duration of W2P by mentor type

For the whole group of women, average maternal EPDS scores decreased significantly over the duration of W2P (*p* = < 0.001). See Table 3. These decreases were statistically significant for women mentored by their own mother (*p* <.001), sister (*p* =.001), or volunteer (*p* =.035), but not statistically significant for women mentored by their mother-in-law, other family, or friend.

**Table 3:** Maternal Mean and Standard Deviations on the Edinburgh Postnatal Depression Scale by Mentor Type at Enrollment and 2 Months and 6 Months Postpartum (*N* = 312)

	Mother *n* = 93	Mother-in-Law *n* = 16	Sister *n* = 23	Other Family *n* = 30	Friend *n* = 102	Volunteer *n* = 48		Full Sample *N* = 312
EPDS Score	*M (SD)*	*M (SD)*	*M (SD)*	*M (SD)*	*M (SD)*	*M (SD)*	*p* ^b^	*M (SD)*
3rd trimester	5.24 (3.36)	7.44 (3.54)	5.04 (3.38)	5.83 (2.85)	5.79 (4.62)	6.27 (4.81)	0.254	5.73 (4.03)
2 months postpartum	4.01 (2.93)	5.44 (3.18)	3.44 (3.03)	4.87 3.20)	5.20 (4.25)	5.33 (4.40)	0.160	4.72 (3.72)
6 months postpartum	3.63 (3.38)	6.00 (3.35)	2.22 (1.86)	4.57 (3.80)	5.00 (4.32)	5.02 (4.09)	**0.002**	4.40 (3.87)
*p* ^a^	**< 0.001**	0.097	**0.001**	0.058	0.133	**0.035**		**< 0.001**
Median differences ^a^ (paired)	-2.00	-2.00	-3.00	-1.50	0.00	-1.00		-1.00

*Note*. EPDS = Edinburgh Postnatal Depression Scale. ^a^ between Enrollment and 6 Months postpartum, ^b^ Kruskal-Wallis test; significant *p*-values in bold

### Comparison of trajectories of women’s depressive symptoms by mentor type

**Fig. 1:**
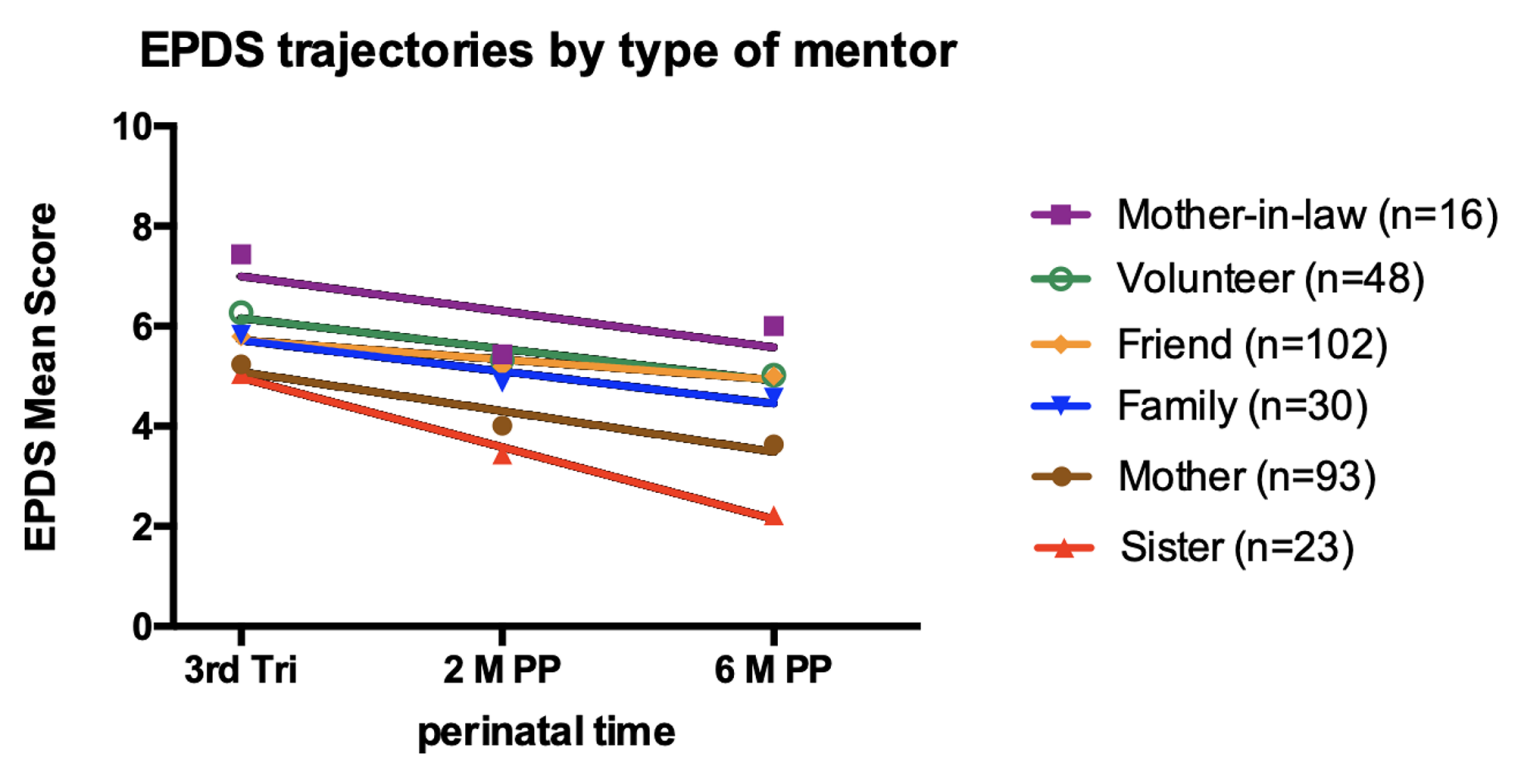
*Note*. Comparison of EPDS score trajectories of first-time mothers over the duration of Welcome to Parenthood intervention by mentor type (*N* = 312). EPDS = Edinburgh Postnatal Depression Scale; 3rd Tri = third trimester; 2 M PP = 2 months postpartum; 6 M PP = 6 months postpartum. Circles, triangles, diamonds and squares represent average EPDS scores at each time point. Elevations of the trajectories are significantly different by mentor type (*p* <.001). Slopes of the trajectories are not significantly different by mentor type

To compare trajectories of women’s depressive symptoms, we fitted the average EPDS scores to straight lines, one for each mentor type (Fig. 1). The elevations (Y-intercepts), slopes, and goodness-of-fit of fitted lines are presented in Table 4.

**Table 4:** Tabular results for EPDS trajectories fitting

		Mother	Mother-in- Law	Sister	Other Family	Friend	Volunteer
		Best-fit value (*SD)* [95% CI]	Best-fit value (*SD)* [95% CI]	Best-fit value (*SD)* [95% CI]	Best-fit value (*SD)* [95% CI]	Best-fit value (*SD)* [95% CI]	Best-fit value (*SD)* [95% CI]
**Elevation** **(Y-intercept when X = 0.0)**	***	5.09 (0.33) [0.93, 9.25]	7.00 (0.95) [-5.22, 19.22]	4.97 (0.17) [2.86, 7.08]	5.71 (0.26) [2.45, 8.98]	5.72 (0.16) [3.76, 7.70]	6.16 (0.24) [3.09, 9.23]
**Slope**	**-**	-0.20 (0.06) [-1.03, 0.62]	-0.18 (0.19) [-2.61, 2.24]	-0.36 (0.03) [-0.78, 0.06]	-0.16 (0.05) [-0.81, 0.49]	-0.10 (0.03) [-0.49, 0.29]	-0.16 (0.05) [-0.77, 0.45]
**Goodness of Fit (** ***R*** ^**2**^ **)**	-	0.91	0.47	0.99	0.91	0.91	0.92

*Note*. EPDS = Edinburgh Postnatal Depression Scale. *CI* = confidence intervals, *SD* = standard deviation,

*** *p* <.001

The *elevation* of a trajectory illustrates the level of average EPDS scores by mentor type at all time points. The trajectories were visibly stratified (i.e., they did not intersect). For all groups except those mentored by mothers-in-law, EPDS scores had a good fit to the straight line (*R*^2^ > 0.90). There were statistically significant differences, *F*(DFn, DFd) = 11.52 [5, 11], *p* =.0004, in elevations of the fitted lines (Y-intercepts). Women mentored by their mother-in-law had the highest elevations of EPDS score trajectories, and women mentored by their sisters had the lowest. The elevation of EPDS scores for the remaining mentor types fell between the levels of mother-in-law and sister.

The *slope* of a trajectory illustrates the rate of decline of EPDS mean scores by mentor type. The slope of the line for EPDS mean scores for the sister mentor type was the steepest; for the friend mentor type, it was the flattest. Women who were mentored by their sisters reported the greatest decrease in depressive symptoms, and those mentored by friends reported the least decrease in depressive symptoms over the duration of W2P. There were no statistically significant differences in the slopes of the lines for EPDS mean scores across mentor type, *F*(DFn, DFd) = 0.98 [5, 6], *p* =.4984. That is, there was no difference in the rate of decrease of depressive symptoms by mentor type over the duration of W2P.

## Discussion

Our single-group, longitudinal study of W2P for first-time mothers adds to the growing body of literature showing that social support is beneficial for perinatal mental health and how outcomes vary by relationship. Social support provided by the women’s mother or sister was associated with the lowest levels of depressive symptoms and with the most significant reduction in depressive symptoms. Social support provided by community volunteers was also associated with a significant reduction in depressive symptoms, despite higher initial depressive symptom levels than in close family mentored groups. Our results are consistent with a recent narrative review and meta-analysis of 11 studies in which Riem et al. [17] concluded that high-quality involvement from the infant’s grandparents, especially from maternal grandmothers, was associated with better maternal mental health. Similarly, in a systematic review of 13 studies of biological and psychosocial predictors of PPD, Yim at al [23]. concluded that support from a woman’s family, especially her mother, may have a protective influence against PPD. Another study also reported that support from women’s parents was correlated with better mood [24]; and in Türkiye, women’s own mothers were key providers of support [25]. Our results are also in line with traditional perinatal support practices in which mostly the closely related women are involved [26].

To our knowledge, there are no other studies investigating the role of social support provided by sisters in perinatal mental health. A better understanding of the role of sisters may be important for designing future interventions to support women who are first-time mothers in the transition to parenthood. Our finding that social support provided by community volunteers was associated with fewer depressive symptoms is consistent with studies showing that structured social support provided by community volunteers significantly improved mothers’ emotional well-being and self-esteem in first-time disadvantaged mothers [27, 28].

Our result that mother and sister mentor types were associated with the lowest depressive symptoms and significant decreases in depressive symptoms aligns with Stewart’s [10] social support theory. Maternal appraisal of social embeddedness, durability, and stability of support from mothers and sisters may contribute to better mental health. Support from mothers and sisters may be perceived as stable as they are embedded in close family relationships. Generalized reciprocity may play a role [29]. The woman may feel less indebted towards her closest family, as she will be able to return the support later. The result that depressive symptoms decreased significantly for women mentored by volunteers also aligns well with the theory. Community volunteers may be more committed to providing regular support than friends or coworkers. Moreover, formal training and agreements create clarity around unidirectionality of support without expectation of reciprocity. It remains unclear why social support from mothers-in-law, other family, and friends was not associated with decreases in depressive symptoms. It is possible that lack of social embeddedness and durability of these relationships, along with unclear expectations about reciprocity, may influence the perceived quality of social support. Design of future interventions to decrease postpartum depressive symptoms should assess women’s perceptions of social support by mentor type.

Women’s migratory status and language spoken at home were associated with mentor type. In a narrative review of mechanisms underlying social patterning of PPD in immigrant women, Saad [30] reported that loss of previous support networks and challenges forming new networks after immigration may underlie increased rates of postnatal depression among immigrant women. This mechanism may apply to women who relocate within a country and lose previous social networks and sense of community belonging. In our study, migration and speaking a language other than English at home were associated with being mentored by a friend or volunteer, rather than family.

Although we collected a large sample from a wide geographical area, this was a single group, longitudinal study of W2P without a control group. Given the potential for selection bias and relatively high levels of income and education at the time of this study, the results may not be generalizable to other jurisdictions. Also, we did not collect data about other types of social support women received outside of W2P that may have influenced their depressive symptoms.

The ubiquitous presence of postnatal care practices in contemporary non-Western cultures [26] and in pre 1950s Western world [31], together with growing evidence that support from the child’s maternal grandmother is beneficial for postpartum mental health points to the importance of family members beyond a woman’s partner during the transition to parenthood. Therefore, interventions that facilitate and encourage support from a woman’s family may be helpful in ensuring the preservation of maternal mental health and a successful transition to parenthood. For women who are first-time mothers, education about the importance of social support during this transition, as well as practical advice on how to seek and mobilize such support from one’s own family should be added to prenatal class curricula and to educational booklets. When there is no family support available, women can benefit from support provided by committed community volunteers. To increase consistency with current evidence about support, women, their partners, and mentors need to be educated together to hear the same information about (a) how to support a new mother, (b) risk of PPD, and (c) community resources if the mentor recognizes a concern. Additionally, policies that enable caregivers within the broader family system to participate actively in caring for the new mother and infant (e.g., grandparental leave) should be considered to reduce risk of postnatal depressive symptoms in first-time mothers. The results of this study suggest that informal social support during the transition from pregnancy to postpartum is important. Future research needs to focus on systems in which women seek and use support in the transition from pregnancy to parenthood.

## Data Availability

Data and meta-data are available through Policy Wise, Secondary Analysis to Generate Evidence (SAGE).
